# Direct Comparison of Flow-FISH and qPCR as Diagnostic Tests for Telomere Length Measurement in Humans

**DOI:** 10.1371/journal.pone.0113747

**Published:** 2014-11-19

**Authors:** Fernanda Gutierrez-Rodrigues, Bárbara A. Santana-Lemos, Priscila S. Scheucher, Raquel M. Alves-Paiva, Rodrigo T. Calado

**Affiliations:** 1 Department of Internal Medicine, University of São Paulo at Ribeirão Preto School of Medicine, Ribeirão Preto, São Paulo, Brazil; 2 Center for Cell-based Therapy, São Paulo Research Foundation (FAPESP), Ribeirão Preto, São Paulo, Brazil; Tulane University Health Sciences Center, United States of America

## Abstract

Telomere length measurement is an essential test for the diagnosis of telomeropathies, which are caused by excessive telomere erosion. Commonly used methods are terminal restriction fragment (TRF) analysis by Southern blot, fluorescence *in situ* hybridization coupled with flow cytometry (flow-FISH), and quantitative PCR (qPCR). Although these methods have been used in the clinic, they have not been comprehensively compared. Here, we directly compared the performance of flow-FISH and qPCR to measure leukocytes' telomere length of healthy individuals and patients evaluated for telomeropathies, using TRF as standard. TRF and flow-FISH showed good agreement and correlation in the analysis of healthy subjects (R^2^ = 0.60; p<0.0001) and patients (R^2^ = 0.51; p<0.0001). In contrast, the comparison between TRF and qPCR yielded modest correlation for the analysis of samples of healthy individuals (R^2^ = 0.35; p<0.0001) and low correlation for patients (R^2^ = 0.20; p = 0.001); Bland-Altman analysis showed poor agreement between the two methods for both patients and controls. Quantitative PCR and flow-FISH modestly correlated in the analysis of healthy individuals (R^2^ = 0.33; p<0.0001) and did not correlate in the comparison of patients' samples (R^2^ = 0.1, p = 0.08). Intra-assay coefficient of variation (CV) was similar for flow-FISH (10.8±7.1%) and qPCR (9.5±7.4%; p = 0.35), but the inter-assay CV was lower for flow-FISH (9.6±7.6% vs. 16±19.5%; p = 0.02). Bland-Altman analysis indicated that flow-FISH was more precise and reproducible than qPCR. Flow-FISH and qPCR were sensitive (both 100%) and specific (93% and 89%, respectively) to distinguish very short telomeres. However, qPCR sensitivity (40%) and specificity (63%) to detect telomeres below the tenth percentile were lower compared to flow-FISH (80% sensitivity and 85% specificity). In the clinical setting, flow-FISH was more accurate, reproducible, sensitive, and specific in the measurement of human leukocyte's telomere length in comparison to qPCR. In conclusion, flow-FISH appears to be a more appropriate method for diagnostic purposes.

## Introduction

Telomeres are DNA-protein structures consisted of tandem TTAGGG repeats coated by proteins (shelterin) that cap the ends of linear chromosomes [1]. Telomeres prevent the recognition of normal chromosome ends as double-strand DNA breaks and avoid chromosome instability and the activation of the DNA damage response (DDR) machinery. In humans, telomere shortening has been widely investigated for its involvement in aging and in the development of several diseases (referred to as telomeropathies), including bone marrow failure syndromes (dyskeratosis congenita and aplastic anemia), hepatic cirrhosis, idiopathic pulmonary fibrosis, and cancer susceptibility [2]. Congenital mutations in genes involved in telomere biology lead to excessive telomere shortening, premature cell proliferation arrest, and deficient tissue regenerative capacity. In the clinic, telomere length measurement is used for the diagnosis of telomere diseases and to identify mutation carriers in affected families [3]. Additionally, telomere length measurement is an important tool to investigate telomere biology and the contribution of telomere dysfunction to degenerative disorders.

Several methods are available to measure telomere length [4], [5]. The majority of studies and diagnostic laboratories apply one of the following methods: (1) terminal restriction fragment (TRF) analysis by Southern blot, (2) fluorescence *in situ* hybridization combined with flow cytometry (flow-FISH), or (3) quantitative PCR (qPCR). Quantitative FISH (Q-FISH) and single telomere length analysis (STELA) also are widely employed in non-diagnostic laboratories and are reviewed in [5]. TRF analysis by Southern blot is the standard method, as it directly estimates the average telomere length in kilobases. Described more than 25 years ago [6]–[8], this method measures the average TRF obtained after digestion of genomic DNA with restriction enzymes; telomere length is calculated based on the mobility of electrophoresis-separated TRFs in comparison to known molecular-weight markers. However, TRF analysis has some limitations. It is time-consuming, requires a substantial amount of DNA, may be influenced by “gel effects”, and incorporates the subtelomeric DNA length in the measurement, overestimating the real length of telomeric sequences. Alternative methods, as flow-FISH and qPCR, have become important adjuncts to the more laborious Southern blotting and have the advantage of strictly measuring the canonical telomeric sequences (the TTAGGG repeats).

Flow-FISH is a method that combines fluorescent *in situ* hybridization (FISH) with flow cytometry, using labeled peptide nucleic acid (PNA) probes that hybridize to telomere repeats in cells in suspension [9], [10]. This method estimates the average telomere length based on the average telomere content (quantity of telomere repeats) of single cells, expressed as mean fluorescence intensity and translated into kilobases based on its correlation with TRF analysis. Of note, it allows the measurement of telomere content in specific cell subsets. Flow-FISH has already been validated for clinical purposes [3], [5], [11], but it requires calibration and control for all protocol steps, which makes the whole procedure time-consuming, more expensive, and technically demanding. Additionally, it determines the average telomere content of a given sample and not telomere length.

Real time quantitative PCR (qPCR) has been adapted to measure telomere length by determining the average content of telomere sequences in a given sample using the ratio of telomere repeat copy number from a DNA sample to single copy gene (T/S ratio). It calculates the abundance of telomere sequences in comparison to a genomic single gene by PCR amplification using a double strand DNA-biding dye (SYBR Green) [12]. It has the advantage of being easily performed, requires small amounts of DNA, and is capable of high-throughput analysis of a large numbers of samples. The major limitation is its variance in reproducibility. Differently from Southern blot, flow-FISH and qPCR measure the average telomere content as the mean fluorescence intensity and T/S ratio, respectively, and not length directly in kilobases.

These methodologies employ different tools and use different parameters, often hampering comparisons between studies, and they have not been compared for clinical performances or for whether they are interchangeable. The goal of the present study was to directly compare the clinical use of flow-FISH and qPCR methods as diagnostic tests for telomeres measurement by assessing the reproducibility, accuracy, sensitivity, and specificity of both methods, using the TRF analysis as standard.

## Material and Methods

### Patients and controls

EDTA blood samples were collected from a cohort of 70 healthy individuals and 45 patients with bone marrow failure (BMF) or idiopathic pulmonary fibrosis (IPF) and six family members seen at the University Hospital, University of São Paulo at Ribeirão Preto School of Medicine. Family members were investigated for potential telomere disease; two were later found to carry a *TERT* mutation (R865H) and presented peripheral blood cytopenias. Healthy individuals ranged in age from zero (umbilical cord) to 88 years and patients ranged from seven to 83 years. For each technique, distribution curves were derived from best-fit analysis of telomere length from an independent cohort of at least 180 healthy individuals. The 1st, 10th, 50th, 90th, 99th percentiles were adjusted to the curve **(Figure S1).** Very short telomeres were defined as below the first percentile for age and short telomeres as below the tenth percentile [2], [3], [13]. This study was approved by the local ethics committee (Comitê de Ética em Pesquisa do Hospital das Clínicas de Ribeirão Preto - process number, 12050/2011) and written consent was obtained from all participants or their legal guardians.

### Sample preparation

Three aliquots of nucleated blood cells were separated from each blood sample and telomere length was measured in parallel by Southern blot, flow-FISH, and qPCR. Flow-FISH aliquots were used to isolate the nucleated cells from whole blood. Aliquots were washed in PBS/0.1% BSA and incubated on ice cold NH_4_Cl for osmotic red blood cell lysis. After isolation of white blood cells (WBCs), purified WBCs were counted, aliquoted, and frozen at -80°C in 10% DMSO. Fixed bovine thymocytes (CT), used as internal control, were prepared as described [10]. For Southern blotting and qPCR aliquots, genomic DNA was extracted from buffy coat WBCs by Gentra Puregene Blood kit (Qiagen, Maryland, USA). Buffy coat was isolated by sample centrifugation followed by manual pipetting. DNA samples were quantified and stored at −20°C. For qPCR, DNA dilutions of 50 and 5 ng/µL were prepared and kept frozen. Dilutions of 0.2 ng/µL were used for every run and prepared just before experiments. Genomic DNA (50 ng) was checked for integrity in 1.5% agarose gel at 200 V for 45 min.

### Telomere Fluorescence *In Situ* Hybridization and Flow Cytometry (flow-FISH)

The flow-FISH method was previously described by others [9], [10], [14]–[16]. In our study, for each sample, 8×10^5^ WBCs were divided in four replicates tubes. To control tube-to-tube variation, 10^5^ fixed CT were added to each sample as an internal reference and the Telomere PNA Kit/FITC probe (Dako, Glostrup, Denmark) was used for hybridization, according to manufacturer's instructions. All samples were analyzed in a JSAN flow cytometer (BayBioscences, Kobe, Japan). FITC-labeled fluorescent calibration beads (Quantum FITC-5 MESF; Bangs laboratories, Inc., Indiana, USA) were used to calibrate the flow cytometer and to translate results into standard fluorescence units, as described [16], [17]. Using the Quantum FITC MESF software (Bangs Laboratory), the fluorescence recorded for each sample was converted into equivalent MESF value [10], [15]–[17]. To transform MESF values into kilobases, we utilized the equation described by Kapoor and Telford, 2004 [16]. A reference sample was included as a control in each flow-FISH experiment. All the measurements were normalized with telomere lengths calculated for the CTs added in each tube sample, as previously described [10].

### Southern blot analysis of TRF

Terminal restriction fragment (TRF) analysis was performed according to the manufacturer's instructions with minor changes (TeloTAGGG Telomere Length Assay – Roche Applied Science, Mannheim, Germany). Briefly, genomic DNA (800 ng) was digested by an optimized mixture of *Hinf*I and *Rsa*I FastDigest restriction enzymes (Thermo Scientific, Waltham, MA, USA) at 37°C for 2 h. Following DNA digestion, DNA fragments were separated by electrophoresis in 0.8% agarose gel during 5 h. Gel was denatured, neutralized, and samples were transferred to a nylon membrane by Southern blotting and probed. Terminal restriction fragments were detected by chemiluminescence. Mean TRF length was determined according to the formula TRF = Σ(ODi)/Σ(ODi/Li), where ODi is the chemiluminescent signal and Li is the length of the fragment at a given position. In every experiment, the mean TRF of a reference sample was determined in order to validate the results.

### Telomere length by quantitative PCR (qPCR)

Mean telomere length was measured by qPCR based on a modification of the method described by Cawthon in 2002 [12], as we previously described [18], [19]. Basically, qPCR was conducted in triplicate and reactions included: genomic DNA (1.6 ng), 2x Rotor-Gene SYBR Green, PCR Master Mix (Qiagen, Hilden, Germany), RNase free water (Qiagen), primer Tel Forward (300 nM) (CGGTTTGTTTGGGTTTGGGTTTGGGTTTGGGTTTGGGTT) and Tel Reverse (300 nM) (GGCTTGCCTTACCCTTACCCTTACCCTTACCCTTACCCT) or primer single gene forward (36B4 F–300 nM) (CAGCAAGTGGGAAGGTGTAATCC) and single gene reverse (36B4 R–500 nM) (CCCATTCTATCATCAACGGGTACAA), in a 24 µL final reaction. Sequence primers were previously described by Brouilette *et al*., 2007 [20]. All qPCR reactions were prepared on a QIAgility automated pipettor (Qiagen, California, USA) and amplification was conducted in the Rotor-Gene Q (Qiagen) real-time PCR cycler as follow: 5 min at 95°C, 25 cycles (telomere reaction) and 35 cycles (single gene reaction) of 7 sec at 98°C and 10 sec at 60°C (telomere) or 10 sec at 58°C (single gene). The telomere length for each sample was determined using the telomere to single copy gene ratio (T/S ratio) by calculating the ΔCt [Ct(telomere)/Ct(single gene)]. The T/S ratio for each sample (x) was normalized to the mean T/S ratio of reference sample [2–(ΔCtx−ΔCtr)  = 2−ΔΔCt], which was also used for the standard curve, both as a reference sample and as a validation sample. In every run, two reference samples were included to validate each reaction. The experiment was considered acceptable if control sample T/S ratio ranged within the 95% variation interval (0.95–1.05). The correlation between telomere length measurements of an independent cohort (n = 76) by qPCR and Southern blot was used to convert T/S ratio values in kilobases. The linear regression equation used was: telomere length (kb)  = 4.330x+5.07 (R^2^ = 0.55; p<0.0001), where x corresponds to the T/S ratio value.

### Statistical analysis

As commonly seen in clinical measurement comparisons, linear regression was used to obtain the correlation between telomere length measurements by qPCR, mean TRF length by Southern blot, and flow-FISH. However, as proposed by Bland-Altman in 1986 [21], agreement analysis also was employed, as it is a more appropriate statistical tool to compare clinical assays that measure the same parameter. The bias and limits of agreement (LoA) were compared to assess the performance of all methods, evaluating the agreement and precision between them [22]. Analyses were performed comparing two methods at a time. Bland-Altman analysis also was used to evaluate the reproducibility of flow-FISH and qPCR. The intra-assay and inter-assay coefficients of variation (CV) for each technique were determined. Sensitivity and specificity of flow-FISH and qPCR to detect patients with short and very short telomeres were evaluated in comparison to the standard method (Southern blot). For these analyses, we evaluated two different cut-offs. For the more stringent cut-off, we considered as “positive” patients fulfilling both criteria: (1) clinically diagnosed with dyskeratosis congenita or aplastic anemia bearing a known pathogenic telomerase mutation and (2) telomere lengths below the first percentile by TRF analysis. For the second cut-off, we considered as “positive” patients with telomere lengths below the 10th percentile by TRF analysis. Telomere length of patients was defined as short, very short or normal (above 10th percentile) by Southern blot. Telomere length measurement by flow-FISH and qPCR were compared to Southern blot in order to evaluate the proportion of patients who were correctly identified with short or very short telomeres by these techniques (sensitivity) and the proportion of patients who were correctly identified with normal telomeres (specificity). Statistical data analyses were performed using GraphPad Prism v5 (GraphPad Software Inc, CA, USA) and R software (v3.0.3).

## Results

Seventy healthy individuals and 51 patients with BMF and/or IPF were included in our study. The median age and clinical characteristics of participants are shown in Table 1. Telomere lengths of all the participants were determined by Southern blot, flow-FISH, and qPCR. Linear correlation between TRF analysis, flow-FISH, and T/S ratio and Bland-Altman analysis for agreement of these techniques are shown in Table 2.

**Table 1:** Demographic and clinical characteristics of participants of this study.

	n	Median age (range)
**Healthy subjects**	**70**	**42 (0–88)**
Female	33	36 (1–83)
Male	35	49 (1–88)
Umbilical cord	2	0
**Patients**	**51**	**51 (7–83)**
Aplastic anemia (AA)	26	39 (7**–**81)
Dyskeratosis congenita (DC)	3	12 (9–20)
Idiopathic pulmonary fibrosis (IPF)	16	16 (35–83)
Family members	6	34 (16–59)

**Table 2:** Comparison between telomere length methods: linear regression and Bland-Altman analysis.

	Linear regression			Bland-Altman analysis	
	Equation	R^2^	p value	Bias ±SD	Limits of agreement: lower and upper
**Flow-FISH x TRF**					
Healthy subjects	y = 0.85+0.86x	0.60	<0.0001	0.17±1.03	−1.88/2.24
Patients	y = 2.1+0.67x	0.51	<0.0001	0.0±1.21	−2.41/2.41
**qPCR x TRF**					
Healthy subjects	y = 5.2+3.1x	0.35	<0.0001	0.78±1.34	−1.90/3.47
Patients	y = 4.8+2.8x	0.20	0.001	1.15±1.49	−1.84/4.14
**Flow-FISH x qPCR**					
Healthy subjects	y = 5.6+2.7x	0.33	<0.0001	–0.6±1.27	−3.16/1.94
Patients	y = 5.6+1.2x	0.1	0.08	–1.15±1.65	–4.45/2.15

### Correlation and agreement

**Figure 1:**
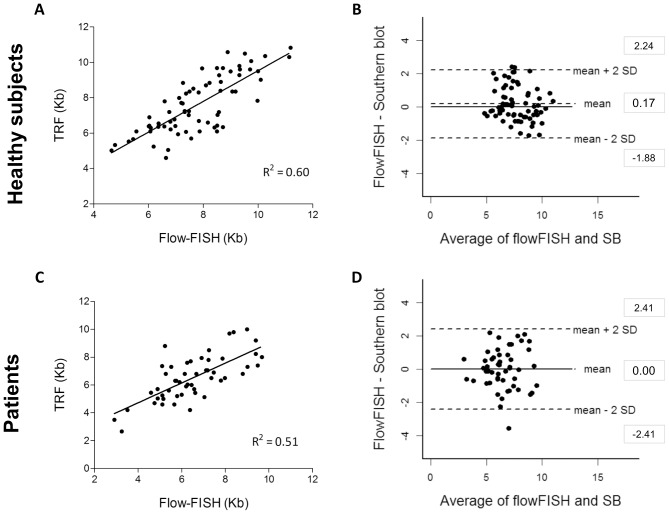
Comparison between flow-FISH and TRF analysis of leukocyte telomere length in healthy individuals and patients with bone marrow failure or idiopathic pulmonary fibrosis: linear correlation and Bland-Altman agreement. Telomere length of 70 healthy individuals was measured by flow-FISH and Southern blot: (**A**) Linear regression plots; solid line represents the data best fit (R^2^ = 0.60); and (**B**) Bland-Altman plot for agreement analysis of flow-FISH (kb) and TRF analysis (kb). The bias±SD was 0.17±1.03 and limits of agreement (LoA) ranged from −1.88 to 2.24 kb. Telomere length of 51 patients was measured by both methods: (**C**) Linear regression plots; solid line represents the data best fit (R^2^ = 0.51); and (**D**) Bland-Altman plot for agreement analysis of flow-FISH (kb) and TRF analysis (kb). The bias±SD was zero ±1.21 and the LoA ranged from −2.41 to 2.41. Measurements were represented in kilobases.

Flow-FISH measurements correlated with TRF analysis for both healthy subjects (R^2^ = 0.60; p<0.0001) and patients (R^2^ = 0.51, p<0.0001) (**Figure 1A and C**). Bland-Altman analysis evidenced good agreement between the two methods (**Figure 1B and D**). The mean difference between flow-FISH and TRF analysis for healthy individuals was 0.17 kb, with the LoA varying from −1.88 kb to 2.24 kb. The bias for the analysis of patients' samples was zero (LoA ranging from −2.41 kb to 2.41 kb).

**Figure 2:**
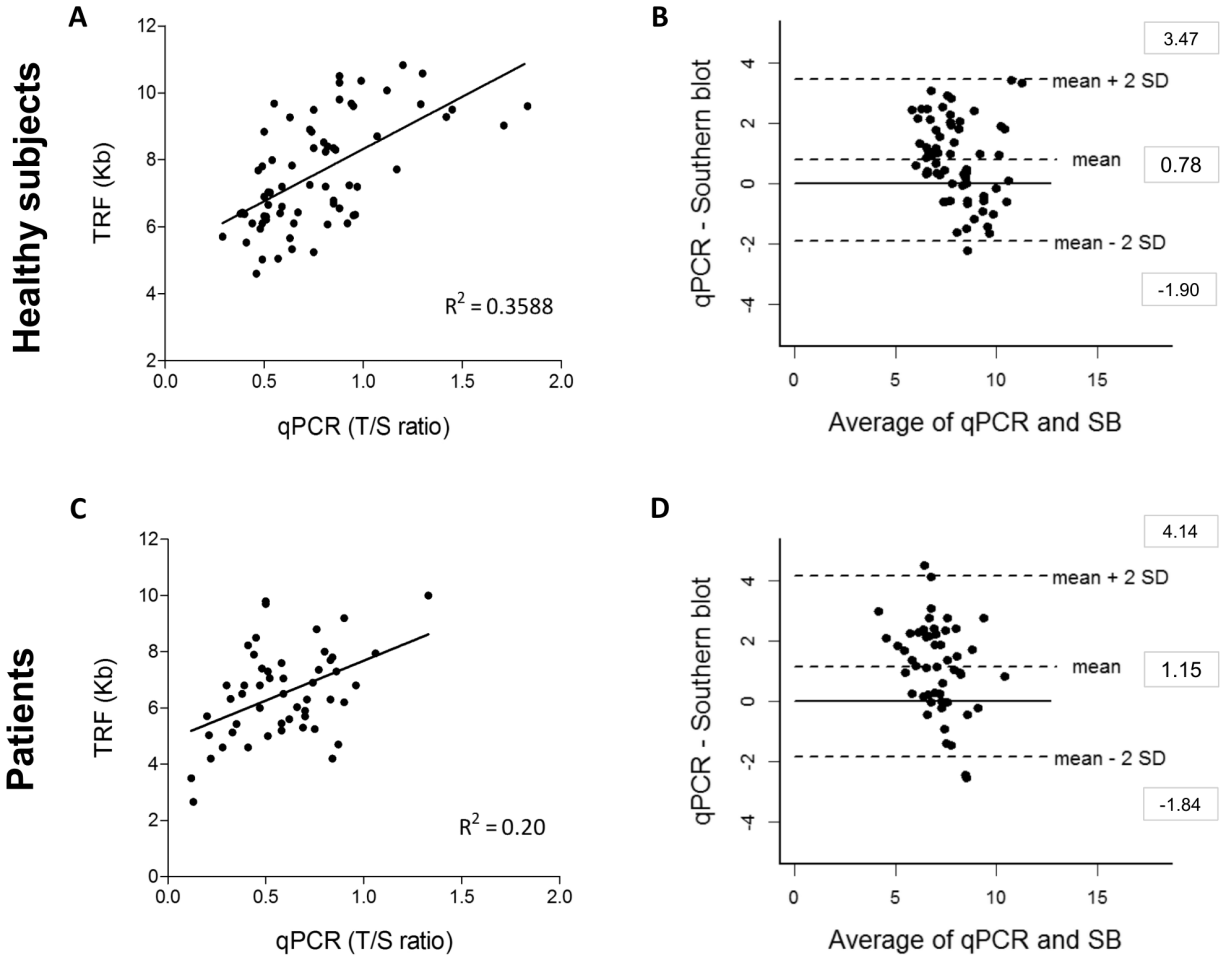
Comparison between qPCR and TRF analysis of leukocyte telomere length in the healthy individuals and patients with bone marrow failure or idiopathic pulmonary fibrosis: linear correlation and Bland-Altman agreement. Telomere length from 70 healthy individuals was measured by qPCR and Southern blot: (**A**) Linear regression plots of qPCR (T/S ratio) x TRF analysis (kb) measurements; solid line represents the data best fit (R^2^ = 0.35); and (**B**) Bland-Altman plot for agreement analysis of qPCR (kb) and TRF analysis (kb). The bias±SD was 0.78±1.34 and limits of agreement (LoA) ranged from −1.90 to 3.47. Telomere length of 51 patients was measured by both methods: (**C**) Linear regression plots qPCR (T/S ratio) x TRF analysis (kb) measurements; solid line represents the data best fit (R^2^ = 0.20) and (**D**) Bland-Altman plot for agreement analysis of qPCR (kb) and TRF analysis (kb). The bias±SD was 1.15±1.49 and the LoA ranged from −1.84 to 4.14.

The comparison between qPCR and Southern blot for the same sets of samples showed more modest correlation, which was lower in patients (R^2^ = 0.20; p = 0.001) than in healthy individuals (R^2^ = 0.35; p<0.0001) (**Figure 2A and C**). Bland-Altman analysis confirmed that the agreement and precision between qPCR and Southern blot was less adequate than for flow-FISH. The biases for healthy subjects and patients were 0.78 kb and 1.15 kb, respectively, and the limits of agreement were larger (**Figure 2B and D**).

**Figure 3:**
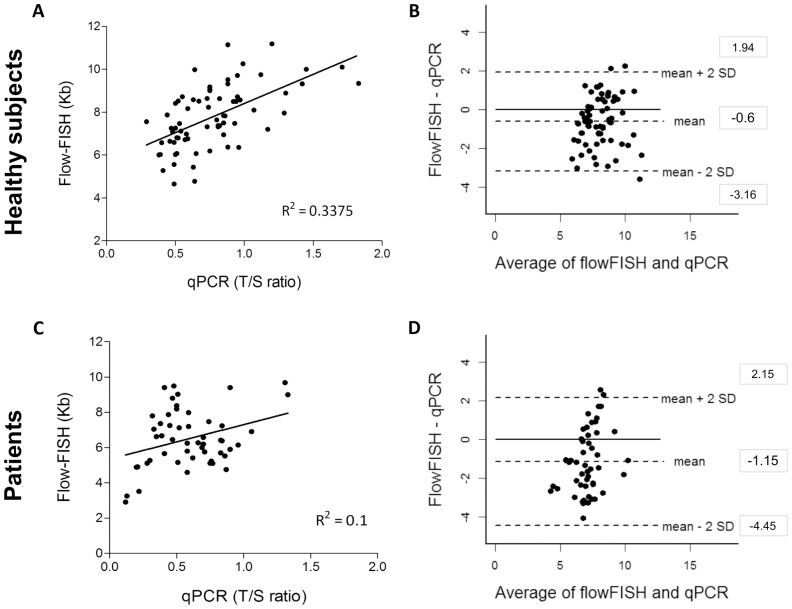
Comparison between qPCR and flow-FISH of leukocyte telomere length in the healthy individuals and patients with bone marrow failure or idiopathic pulmonary fibrosis: linear correlation and Bland-Altman agreement. Telomere length of 70 healthy individuals was measured by qPCR and flow-FISH: (**A**) Linear regression plots of qPCR (T/S ratio) x flow-FISH (kb) measurements; solid line represents the data best fit (R^2^ = 0.33); and (**B**) Bland-Altman plot for agreement analysis of qPCR (kb) and flow-FISH (kb). The bias±SD was -0.6±1.27 and limits of agreement (LoA) ranged from −3.16 to 1.94. Telomere length of 51 patients was measured by both methods: (**C**) Linear regression plots of qPCR (T/S ratio) x flow-FISH (kb) measurements and solid line represents the data best fit (R^2^ = 0.10). (**D**) Bland-Altman plot for agreement analysis of qPCR (kb) and flow-FISH (kb). The bias±SD was −1.15±1.65 and the LoA ranged from −4.45 to 2.15.

We next directly compared flow-FISH and qPCR and found a modest correlation between methods for healthy subjects measurements (R^2^ = 0.33, p<0.0001). However, there was no correlation between qPCR and flow-FISH in the analysis of patients' samples (R^2^ = 0.1, p = 0.08) (**Figure 3A and C**). These findings were endorsed by Bland-Altman analysis: the agreement between these assays for both healthy subjects and patients was poor (**Figure 3B and D**). Mean differences and limits of agreement between flow-FISH and qPCR for healthy individuals was −0.6 kb (LoA, −3.16 kb to 1.94 kb). For patients, the variance of differences also was high, with a mean difference of −1.15 kb (LoA, −4.45 kb to 2.15 kb). These results were similar to those observed when qPCR and TRF were compared.

### Intra-assay variability

**Figure 4:**
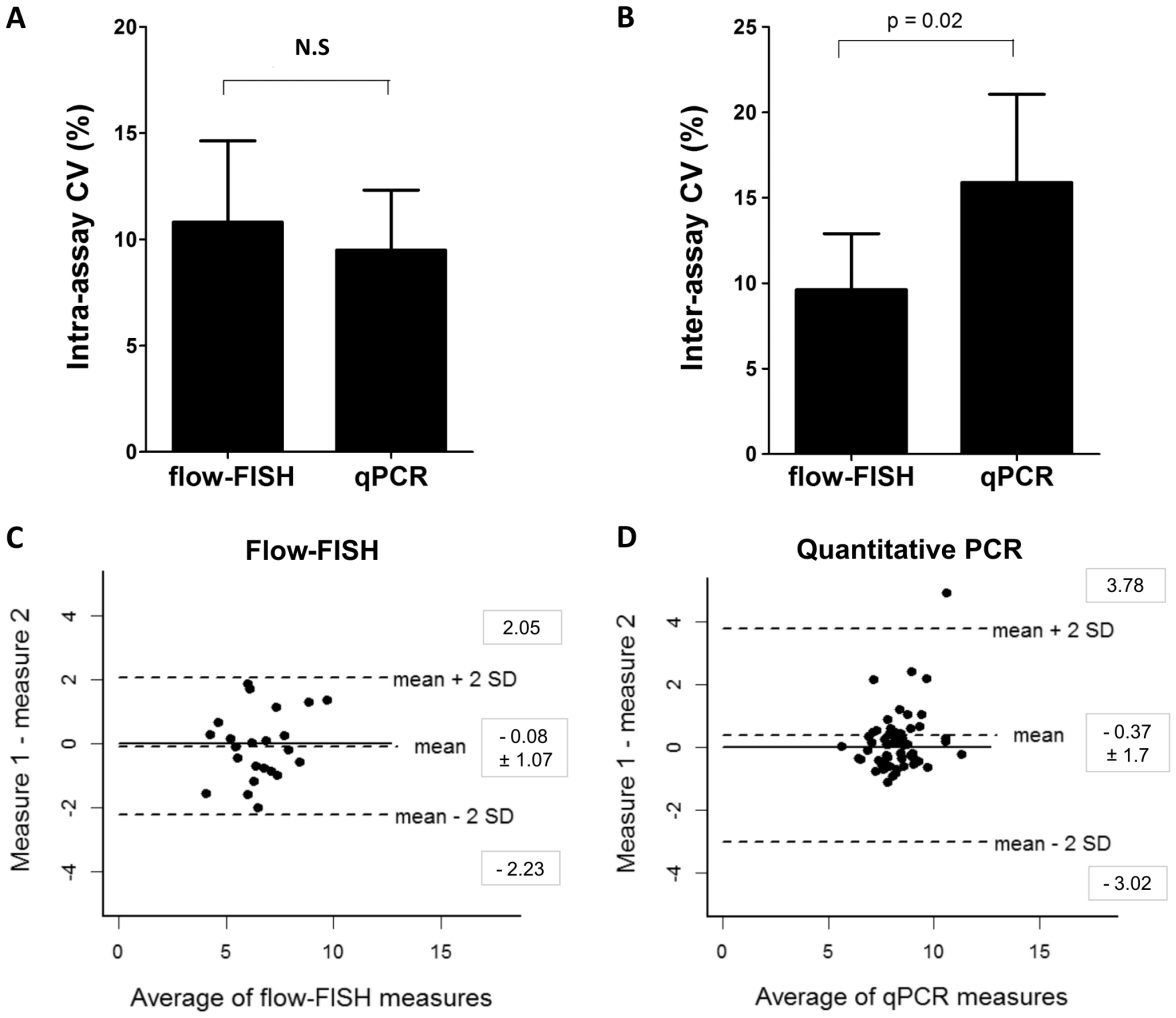
Variability analysis of flow-FISH and qPCR. (**A**) Intra-assay variation ±95% confidence interval. Flow-FISH, CV = 10.8%; qPCR, CV = 9.5%; p = 0.35. (**B**) Inter-assay variation ±95% confidence interval. Flow-FISH, CV = 9.5%; qPCR, CV = 16%; p = 0.02. (**C**) Bland-Altman plot for flow-FISH: mean difference between two independent measurements of 23 samples by their average; the bias was −0.08±1.07 and limits of agreement ranged from −2.23 to 2.05. (**D**) Bland-Altman plot for qPCR: mean difference between two independent measurements of 57 samples by their average; the bias was −0.37±1.7 and limits of agreement ranged from −3.02 to 3.78. For Bland-Altman analysis, telomere length measurements were represented in kilobases.

A reference sample was run in duplicate in each experiment for both flow-FISH and qPCR. The analysis of the variation in telomere length for the reference sample revealed that the intra-assay CV for flow-FISH was 10.8±7.1% and 9.5±7.4% for qPCR, which were not statistically different (p = 0.35) (**Figure 4A**).

### Inter-assay variability

Reproducibility of the standard method Southern blot was determined by analyzing the telomere length result for a reference sample run in all experiments. The inter-assay CV of the TRF analysis in our laboratory was 5.8±5.9%. Similarly, the variability of flow-FISH and qPCR was also determined and compared. The inter-assay CV was lower for flow-FISH (9.6±7.6%) in comparison to qPCR (15.9±19.4%; p = 0.02) (**Figure 4B**). Bland-Altman analysis of the differences between replicate measurements also showed that flow-FISH was more reproducible and more precise (with lower variability) than qPCR: the bias for flow-FISH was −0.08 kb (limits of agreement, −1.15 kb to 0.99 kb; **Figure 4C**), whereas it was higher for qPCR (mean, −0.37 kb; limits of agreement, −3.02 kb to 3.78 kb; **Figure 4D**).

### Sensitivity and specificity

**Figure 5:**
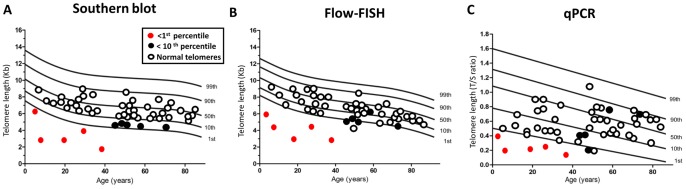
Telomere length according to age in patients with bone marrow failure or idiopathic pulmonary fibrosis measured by Southern blot, flow-FISH and qPCR. Lines represent the 1st, 10th, 50th, 90th, 99th percentiles of telomere length of healthy controls (**Figure S1**). (**A**) Telomere length measurement by Southern blot was used to define patients with dyskeratosis congenita or BMF/IPF with telomerase mutation who had very short telomeres (<1st percentile; represented by red dots), patients with short telomeres (<10th percentile; represented by black dots) and patients with normal telomeres (above 10th percentile; circles). (**B**) Telomere length measurement by flow-FISH of patients with very short, short and normal telomeres defined by Southern blot. (**C**) Telomere length measurement by qPCR of patients with very short, short and normal telomeres defined by Southern blot.

Sensitivity and specificity of flow-FISH and qPCR were evaluated in comparison to the standard method (Southern blot). Telomere length measurement by flow-FISH and qPCR were compared to Southern blot (**Figure 5A–C**). Flow-FISH displayed a sensitivity of 80% and specificity of 85% for distinguishing patients with short telomeres (below 10th percentile). In the detection of very short telomeres (below 1st percentile) in patients with dyskeratosis congenita or BMF/IPF with known telomerase mutations, flow-FISH displayed 100% sensitivity and 95% specificity. Differently, qPCR was less sensitive (40%) and specific (63%) than flow-FISH to discriminate patients with short telomeres. In the detection of patients with very short telomeres, qPCR presented 100% sensitivity and 89% specificity.

## Discussion

In the present study, we demonstrated that flow-FISH was more accurate, precise, and reproducible than qPCR for the measurement of telomere length of human peripheral blood leukocytes of healthy subjects and patients with telomere diseases. Flow-FISH displayed a better correlation and agreement with TRF analysis by Southern blot in comparison to qPCR, suggesting that it may be a better method for diagnostic purposes. Additionally, flow-FISH showed better sensitivity and specificity in the detection of patients with short telomeres. To the best of our knowledge, this is the first study to directly compare the performance of these two methods for the measurement of telomere length. Few studies compared assays for telomere length and none of them assessed the performance of flow-FISH and qPCR directly [23]–[25].

Telomere length measurement is the most cost-effective way to identify patients with BMF or IPF with an underlying telomere-biology disorder [26], [27]. To date, no study has clinically compared methods nor established the ideal test for the diagnosis of telomeropathies. Techniques currently employed have advantages and disadvantages, making each one more appropriate at specific experimental scenarios. Southern blot is the gold-standard assay and it is widely used to calibrate and validate other techniques. The choice between flow-FISH and qPCR depends on the type/amount of material available, laboratory infrastructure, cost, sample size, and the accuracy required for a specific goal.

In our study, peripheral blood leukocyte's telomere length of healthy individuals and patients with BMF and IPF was determined by flow-FISH, qPCR, and Southern blot. Whereas flow-FISH measurements yielded results more closely related to TRF analysis, qPCR showed some shortcomings. The flow-FISH protocol included optimization of all steps, which reduces potential interferences during the procedure. In flow cytometry, it is possible to use a gate strategy that excludes damaged cells and at the same time evaluates a large number of cells. The inclusion of bovine thymocytes (internal control cells) along with leukocytes in each tube sample and the use of appropriate control samples correct potential variations in hybridization and monitor the accuracy of procedure steps [15], [28]. Quantitative PCR also includes a set of controls on every plate, needed to compare results among individual experiments. However, the single-gene primers, which control for amplification variations, are run in a separate reaction and intrinsic variability in pipetting is inevitable. Development of a monochrome multiplex qPCR appears to increase the accuracy and reproducibility [29]. All these factors may explain the differences we observed in accuracy and reproducibility between flow-FISH and qPCR. It is important to note, however, that the variation in the correlation between TRF analysis and the two other methods may also be blamed on Southern blot intrinsic limitations: in our hands, the CV for TRF analysis was 5.8±5.9%. Although this is in agreement with previous observations [23], [24], [30], this variation cannot be neglected and may add, to some extent, to the degree of variability between methods.

Direct comparison between flow-FISH and qPCR displayed, for healthy subjects, an agreement and precision similar to the one found for qPCR and TRF analysis. Although in large-scale studies qPCR may be easy to perform telomere length measurement, in the present work, qPCR did not correlate and showed poor agreement with flow-FISH for patient's samples. It is possible, however, that a sum of experimental variability of flow-FISH, qPCR, and Southern blot may have led to errors in measurements of patients' samples. That flow-FISH agreed with TRF analysis for measuring telomere length in patients suggests that the low correlation and agreement observed were related to qPCR features. A similar study that evaluated a large sample size of elderly individuals found a correlation between qPCR and TRF analysis as low as ours (R^2^ = 0.27) [24]. Although other studies found a better correlation than in the present study, they measured telomere length in DNA samples of healthy individuals only [12], [20], [23], [25]. One explanation for the disparity between previous findings [12] and ours is that we have analyzed a set of consecutive samples and samples were not selected or discarded. It also is important to mention that subsequent studies [20], [25] did not reach the high R^2^ value observed in the original description of the method [12].

The discrepancy of agreement between patients and healthy subject samples may be due to pre-analytical factors or to sample intrinsic features. Quantitative PCR is more susceptible to minor variances in sample conditions and DNA integrity. Although our samples were checked for integrity, storage and sample preparation may have affected DNA quality. Blood collection and DNA purification also might contribute to qPCR variability. Due to the pathophysiology of some telomere diseases, the paucity of cells in patients' samples may interfere on DNA purification and directly affect the quality of genomic DNA. Small variations in sample quality as well as individual donor differences may be amplified by qPCR, ultimately leading to the low accuracy observed in patients' telomere length measurements.

The comparison of our work with previous studies reinforced our findings. Similar to our work, previously reported errors (inter-assay CV) for qPCR ranged from 6.45% to 28% [23], [31], [32]. Only one study reported a qPCR inter-assay CV below 6% [33]. However, for flow-FISH, measurement errors are not commonly informed. One study reported an inter-assay CV of 3.3% for lymphocytes [5]. A limitation of the present study is the number of healthy individual assessed.

Each of the three methods employs different tools that are not equally performed among laboratories, which may generate measurement errors and differences in telomere length. In our study, the three techniques were conducted in the same laboratory, mitigating potential influences of sample handling and shipping, personnel, or protocols. In conclusion, our findings suggest flow-FISH has a better performance in the measurement of telomere length of clinical samples, as compared to qPCR. Flow-FISH accuracy, precision, and reproducibility are crucial when diagnosis is the main goal.

## Supporting Information

Figure S1 Telomere length in peripheral-blood leukocytes from three independent cohorts of healthy subjects. The vertical axis represents telomere length in kilobases and distribution curves were derived from best-fit analysis of telomere length from healthy individuals. Lines represent the first, tenth, 50th, 90th, and 99th percentiles. Each black circle represents the telomere length measurement of an individual. **(A) Southern blot**. Telomere length of 302 healthy subjects according to age. The best-fit model that describes the relationship between age and telomere length measured by Southern blot is a third order polynomial model according to the equation: Telomere length (kb)  = 10.14–0.16x+0.002x^2^–0.00001x^3^; (R^2^ = 0.40). **(B) Flow-FISH.** Telomere length of 180 healthy subjects according to age. The best-fit model that describes the relationship between age and telomere length measured by flow-FISH is a third order polynomial model according to the equation: Telomere length (kb)  = 10.36–0.12x+0.001x^2^–0.00001x^3^; (R^2^ = 0.60). **(C) Quantitative PCR.** Telomere length of 261 healthy subjects according to age. The best-fit model that describes the relationship between age and telomere length measured by qPCR is a linear regression analysis described by the following equation: T/S ratio  = 1.08–0.007x; (R^2^ = 0.32, p<0.0001).
(TIF)

Table S1 Primary data on telomere length measurement in healthy controls and patients by flow-FISH, qPCR and Southern blot.
(PDF)
