# Supplementary figures and images for: Direct Comparison of Flow-FISH and qPCR as Diagnostic Tests for Telomere Length Measurement in Humans
Source: PLoS One. 2014 Nov 19;9(11):e113747. doi: 10.1371/journal.pone.0113747 (PMC4237503; doi:10.1371/journal.pone.0113747)

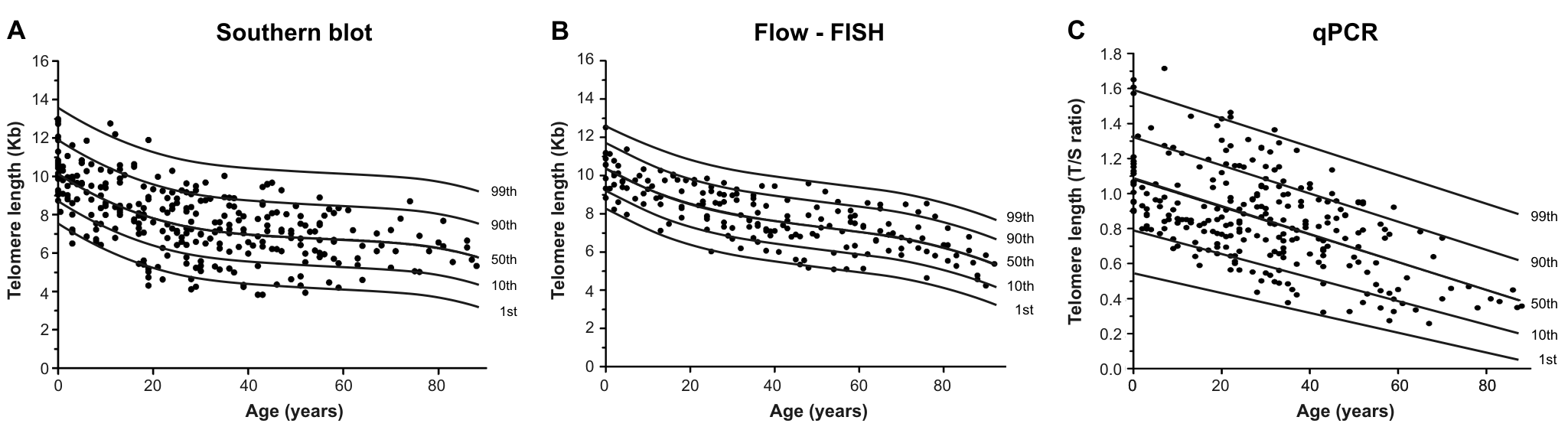

Supplement: Figure S1 — Telomere length in peripheral-blood leukocytes from three independent cohorts of healthy subjects. The vertical axis represents telomere length in kilobases and distribution curves were derived from best-fit analysis of telomere length from healthy individuals. Lines represent the first, tenth, 50th, 90th, and 99th percentiles. Each black circle represents the telomere length measurement of an individual. (A) Southern blot. Telomere length of 302 healthy subjects according to age. The best-fit model that describes the relationship between age and telomere length measured by Southern blot is a third order polynomial model according to the equation: Telomere length (kb) = 10.14–0.16x+0.002x2–0.00001x3; (R2 = 0.40). (B) Flow-FISH. Telomere length of 180 healthy subjects according to age. The best-fit model that describes the relationship between age and telomere length measured by flow-FISH is a third order polynomial model according to the equation: Telomere length (kb) = 10.36–0.12x+0.001x2–0.00001x3; (R2 = 0.60). (C) Quantitative PCR. Telomere length of 261 healthy subjects according to age. The best-fit model that describes the relationship between age and telomere length measured by qPCR is a linear regression analysis described by the following equation: T/S ratio = 1.08–0.007x; (R2 = 0.32, p<0.0001). (TIF) [file pone.0113747.s001.tif]
