# Supplementary material for: Direct Comparison of Flow-FISH and qPCR as Diagnostic Tests for Telomere Length Measurement in Humans
Source: PLoS One. 2014 Nov 19;9(11):e113747. doi: 10.1371/journal.pone.0113747 (PMC4237503; doi:10.1371/journal.pone.0113747)
Supplement: Table S1 — Primary data on telomere length measurement in healthy controls and patients by flow-FISH, qPCR and Southern blot. (PDF) [file pone.0113747.s002.pdf]

Supplementary Table S1. Primary data on telomere length measurement in healthy controls and patients by flow-FISH, qPCR and Southern blot.

ID Samples	Group	Age	Sex	flowFISH(kb)	TRF(kb)	qPCR (T/S ratio)	qPCR (kb)	Diagnosis
C1	Control	0	Umbilical Cord	9,32	9,28	1,42	11,17	Healthy
C2	Control	0	Umbilical Cord	11,19	10,83	1,20	10,24	Healthy
C3	Control	13	F	10,26	10,36	0,99	9,35	Healthy
C4	Control	13	M	8,89	10,58	1,30	10,67	Healthy
C5	Control	14	F	9,75	10,07	1,12	9,90	Healthy
C6	Control	13	F	8,50	9,68	0,94	9,12	Healthy
C7	Control	13	M	7,12	7,99	0,54	7,41	Healthy
C8	Control	2	M	8,23	8,84	0,74	8,28	Healthy
C9	Control	5	M	7,96	9,66	1,29	10,61	Healthy
C10	Control	16	M	9,52	10,50	0,88	8,85	Healthy
C11	Control	4	F	9,71	9,60	0,95	9,18	Healthy
C12	Control	9	M	8,51	6,43	0,67	7,97	Healthy
C13	Control	9	M	9,06	8,35	0,75	8,30	Healthy
C14	Control	1	M	9,32	9,80	0,88	8,88	Healthy
C15	Control	1	F	11,14	10,30	0,88	8,87	Healthy
C16	Control	2	M	10,00	9,50	1,45	11,31	Healthy
C17	Control	5	F	9,34	9,60	1,83	12,93	Healthy
C18	Control	2	F	10,10	9,02	1,71	12,44	Healthy
C19	Control	24	F	7,83	8,30	0,86	8,79	Healthy
C20	Control	36	M	7,37	6,07	0,82	8,62	Healthy
C21	Control	43	M	8,72	6,34	0,95	9,18	Healthy
C22	Control	67	M	7,47	7,02	0,53	7,34	Healthy
C23	Control	59	M	7,50	8,35	0,85	8,74	Healthy
C24	Control	56	M	7,08	5,94	0,48	7,14	Healthy
C25	Control	28	F	7,32	7,25	0,73	8,22	Healthy
C26	Control	34	F	7,32	7,20	0,81	8,57	Healthy
C27	Control	18	F	9,18	8,35	0,75	8,29	Healthy
C28	Control	47	M	5,56	6,10	0,49	7,18	Healthy

C29	Control	61	M	7,37	8,24	0,81	8,57	Healthy
C30	Control	64	M	6,64	4,60	0,46	7,05	Healthy
C31	Control	46	M	6,81	6,21	0,51	7,26	Healthy
C32	Control	41	M	6,01	6,39	0,38	6,72	Healthy
C33	Control	27	M	8,52	7,03	0,52	7,33	Healthy
C34	Control	49	M	6,95	6,78	0,85	8,72	Healthy
C35	Control	58	M	7,56	5,70	0,29	6,31	Healthy
C36	Control	62	M	8,57	7,19	0,97	9,24	Healthy
C37	Control	87	M	5,44	5,66	0,63	7,78	Healthy
C38	Control	18	F	9,98	7,83	0,64	7,84	Healthy
C39	Control	29	F	9,00	9,49	0,75	8,30	Healthy
C40	Control	26	F	8,58	9,27	0,63	7,77	Healthy
C41	Control	64	F	6,58	6,38	0,40	6,78	Healthy
C42	Control	38	F	8,41	6,32	0,50	7,23	Healthy
C43	Control	31	M	8,72	9,68	0,55	7,45	Healthy
C44	Control	38	F	7,20	7,71	1,17	10,12	Healthy
C45	Control	25	M	6,03	6,42	0,39	6,76	Healthy
C46	Control	43	F	7,64	8,52	0,80	8,51	Healthy
C47	Control	28	F	8,63	8,40	0,82	8,58	Healthy
C48	Control	36	F	7,48	7,24	0,93	9,06	Healthy
C49	Control	81	F	7,25	7,68	0,47	7,09	Healthy
C50	Control	32	F	7,26	8,84	0,50	7,23	Healthy
C51	Control	86	M	6,08	6,30	0,51	7,29	Healthy
C52	Control	78	M	7,87	6,10	0,44	6,95	Healthy
C53	Control	71	F	8,50	6,10	0,92	9,02	Healthy
C54	Control	88	M	4,78	5,33	0,64	7,81	Healthy
C55	Control	80	M	6,37	6,55	0,88	8,85	Healthy
C56	Control	86	M	6,80	6,65	0,52	7,32	Healthy
C57	Control	81	M	6,36	6,36	0,96	9,20	Healthy
C58	Control	75	F	6,72	5,05	0,57	7,54	Healthy
C59	Control	55	F	8,64	8,90	0,73	8,22	Healthy
C60	Control	70	M	6,76	7,20	0,59	7,63	Healthy

C61	Control	58	F	8,17	6,60	0,59	7,62	Healthy
C62	Control	83	F	5,28	5,53	0,41	6,85	Healthy
C63	Control	68	F	6,98	6,40	0,58	7,57	Healthy
C64	Control	76	M	6,03	6,90	0,50	7,24	Healthy
C65	Control	76	F	4,65	5,02	0,49	7,18	Healthy
C66	Control	72	F	6,59	7,80	0,49	7,20	Healthy
C67	Control	67	F	6,07	6,10	0,65	7,89	Healthy
C68	Control	58	F	7,84	6,69	0,85	8,73	Healthy
C69	Control	70	M	6,19	5,24	0,75	8,31	Healthy
C70	Control	74	F	8,10	8,70	1,07	9,68	Healthy
P1	patient	54	F	7,36	6,50	0,38	6,71	Family member
P2	patient	32	F	7,80	6,32	0,32	6,47	Family member
P3	patient	37	M	7,23	7,80	0,84	8,70	Family member
P4	patient	59	M	6,38	4,20	0,84	8,71	Family member
P5	patient	7	M	9,02	7,30	0,51	7,29	Aplastic anemia
P6	patient	9	F	6,67	6,80	0,39	6,77	Dysketarosis congenita
P7	patient	61	F	7,12	7,06	0,52	7,31	Aplastic anemia
P8	patient	42	F	7,04	5,13	0,33	6,50	Aplastic anemia
P9	patient	55	F	4,89	5,71	0,20	5,95	Aplastic anemia
P10	patient	60	F	7,25	8,50	0,45	7,03	Aplastic anemia
P11	patient	54	F	6,00	5,30	0,69	8,07	Aplastic anemia
P12	patient	28	F	6,45	6,00	0,47	7,11	Aplastic anemia
P13	patient	19	M	9,40	8,23	0,41	6,84	Aplastic anemia
P14	patient	80	M	4,90	5,03	0,21	5,99	Aplastic anemia
P15	patient	35	M	2,91	3,50	0,12	5,58	Idiopathic pulmonary fibrosis
P16	patient	49	F	5,16	5,00	0,51	7,30	Aplastic anemia
P17	patient	27	M	8,38	9,80	0,50	7,26	Aplastic anemia
P18	patient	35	F	8,19	9,70	0,50	7,26	Aplastic anemia
P19	patient	15	M	7,19	7,05	0,59	7,64	Aplastic anemia
P20	patient	58	M	6,62	5,43	0,35	6,60	Idiopathic pulmonary fibrosis
P21	patient	53	M	5,70	6,30	0,83	8,65	Idiopathic pulmonary fibrosis
P22	patient	43	M	5,66	4,60	0,41	6,85	Aplastic anemia

P23	patient	19	M	7,87	7,90	0,44	6,98	Aplastic anemia
P24	patient	67	M	5,27	6,80	0,30	6,36	Aplastic anemia
P25	patient	36	F	6,27	6,03	0,66	7,91	Aplastic anemia
P26	patient	81	F	5,90	6,20	0,90	8,97	Aplastic anemia
P27	patient	22	M	8,80	6,80	0,47	7,09	Aplastic anemia
P28	patient	61	M	5,79	5,20	0,58	7,57	Idiopathic pulmonary fibrosis
P29	patient	79	M	6,14	6,80	0,96	9,22	Idiopathic pulmonary fibrosis
P30	patient	70	F	5,41	5,60	0,62	7,77	Idiopathic pulmonary fibrosis
P31	patient	66	M	5,11	5,25	0,75	8,33	Idiopathic pulmonary fibrosis
P32	patient	58	F	6,20	5,90	0,70	8,12	Idiopathic pulmonary fibrosis
P33	patient	72	M	4,75	4,70	0,87	8,83	Idiopathic pulmonary fibrosis
P34	patient	51	F	4,59	5,45	0,58	7,59	Idiopathic pulmonary fibrosis
P35	patient	26	M	9,68	8,00	1,31	10,76	Aplastic anemia
P36	patient	27	M	9,40	9,20	0,90	8,98	Aplastic anemia
P37	patient	26	M	9,49	7,40	0,48	7,17	Aplastic anemia
P38	patient	70	M	6,25	7,60	0,58	7,58	Idiopathic pulmonary fibrosis
P39	patient	72	F	6,41	7,70	0,83	8,65	Idiopathic pulmonary fibrosis
P40	patient	12	M	3,51	4,20	0,22	6,03	Dysketarosis congenita
P41	patient	83	F	5,10	7,36	0,77	8,41	Idiopathic pulmonary fibrosis
P42	patient	65	M	5,23	8,80	0,76	8,36	Idiopathic pulmonary fibrosis
P43	patient	80	F	6,57	5,70	0,70	8,11	Idiopathic pulmonary fibrosis
P44	patient	57	M	6,91	7,94	1,06	9,67	Idiopathic pulmonary fibrosis
P45	patient	53	F	5,75	6,30	0,71	8,15	Aplastic anemia
P46	patient	22	M	5,52	7,30	0,86	8,80	Aplastic anemia
P47	patient	47	F	7,47	6,90	0,74	8,28	Aplastic anemia
P48	patient	54	M	9,00	10,00	1,33	10,82	Aplastic anemia
P49	patient	27	M	5,11	4,60	0,28	6,28	Family member
P50	patient	16	F	7,98	6,50	0,59	7,64	Family member
P51	patient	20	F	3,25	2,66	0,13	5,66	Dysketarosis congenita

Supplementary Table S2. Results on replicate measurements by flow-FISH and quantitative PCR (qPCR).

ID Sample	Telomere length Measurement	First duplicate	Second duplicate
F1	flow-FISH (kb)	6,39	7,15
F2	flow-FISH (kb)	7,84	7,6
F3	flow-FISH (kb)	5,32	5,76
F4	flow-FISH (kb)	8,15	8,73
F5	flow-FISH (kb)	6,9	7,88
F6	flow-FISH (kb)	6,7	7,55
F7	flow-FISH (kb)	10,4	9,03
F8	flow-FISH (kb)	6,9	6,8
F9	flow-FISH (kb)	7,9	6,76
F10	flow-FISH (kb)	9,5	8,2
F11	flow-FISH (kb)	6,97	5,1
F12	flow-FISH (kb)	6,95	5,25
F13	flow-FISH (kb)	6,06	6,76
F14	flow-FISH (kb)	6,2	6,17
F15	flow-FISH (kb)	7,8	7,98
F16	flow-FISH (kb)	5,2	6,8
F17	flow-FISH (kb)	5,7	6,87
F18	flow-FISH (kb)	4,96	4,3
F19	flow-FISH (kb)	5,4	5,5
F20	flow-FISH (kb)	5,5	7,5
F21	flow-FISH (kb)	3,29	4,84
F22	flow-FISH (kb)	5,30	5,13
F23	flow-FISH (kb)	4,38	4,10
Q1	qPCR (T/S ratio)	1,42	1,47
Q2	qPCR (T/S ratio)	0,99	1,14
Q3	qPCR (T/S ratio)	1,30	1,23
Q4	qPCR (T/S ratio)	1,12	0,88
Q5	qPCR (T/S ratio)	0,74	0,88

Q6	qPCR (T/S ratio)	1,29	1,24
Q7	qPCR (T/S ratio)	0,75	0,72
Q8	qPCR (T/S ratio)	0,88	0,93
Q9	qPCR (T/S ratio)	0,51	0,67
Q10	qPCR (T/S ratio)	0,82	0,72
Q11	qPCR (T/S ratio)	0,53	0,42
Q12	qPCR (T/S ratio)	0,85	0,97
Q13	qPCR (T/S ratio)	0,48	0,44
Q14	qPCR (T/S ratio)	0,73	0,23
Q15	qPCR (T/S ratio)	0,81	0,8
Q16	qPCR (T/S ratio)	0,75	0,68
Q17	qPCR (T/S ratio)	0,51	0,63
Q18	qPCR (T/S ratio)	0,52	0,64
Q19	qPCR (T/S ratio)	0,29	0,38
Q20	qPCR (T/S ratio)	0,97	0,73
Q21	qPCR (T/S ratio)	0,63	0,61
Q22	qPCR (T/S ratio)	0,75	0,79
Q23	qPCR (T/S ratio)	0,63	0,82
Q24	qPCR (T/S ratio)	0,40	0,42
Q25	qPCR (T/S ratio)	1,17	0,62
Q26	qPCR (T/S ratio)	0,47	0,39
Q27	qPCR (T/S ratio)	0,50	0,76
Q28	qPCR (T/S ratio)	0,92	1,02
Q29	qPCR (T/S ratio)	0,88	0,96
Q30	qPCR (T/S ratio)	0,96	0,82
Q31	qPCR (T/S ratio)	0,73	0,59
Q32	qPCR (T/S ratio)	0,59	0,67
Q33	qPCR (T/S ratio)	0,41	0,59
Q34	qPCR (T/S ratio)	0,65	0,82
Q35	qPCR (T/S ratio)	0,85	0,91
Q36	qPCR (T/S ratio)	0,75	0,83
Q37	qPCR (T/S ratio)	0,66	0,64

Q38	qPCR (T/S ratio)	0,90	0,62
Q39	qPCR (T/S ratio)	0,58	0,45
Q40	qPCR (T/S ratio)	0,62	0,57
Q41	qPCR (T/S ratio)	0,70	0,60
Q42	qPCR (T/S ratio)	0,58	0,72
Q43	qPCR (T/S ratio)	1,31	0,80
Q44	qPCR (T/S ratio)	0,48	0,58
Q45	qPCR (T/S ratio)	0,58	0,79
Q46	qPCR (T/S ratio)	0,83	0,77
Q47	qPCR (T/S ratio)	2,71	0,22
Q48	qPCR (T/S ratio)	0,77	0,66
Q49	qPCR (T/S ratio)	0,76	0,69
Q50	qPCR (T/S ratio)	0,70	0,61
Q51	qPCR (T/S ratio)	1,06	0,90
Q52	qPCR (T/S ratio)	1,85	0,71
Q53	qPCR (T/S ratio)	0,86	0,84
Q54	qPCR (T/S ratio)	0,74	0,53
Q55	qPCR (T/S ratio)	0,28	0,36
Q56	qPCR (T/S ratio)	0,59	0,65
Q57	qPCR (T/S ratio)	0,13	0,13
